# Increased survival in puppies affected by Canine Parvovirus type II using an immunomodulator as a therapeutic aid

**DOI:** 10.1038/s41598-021-99357-y

**Published:** 2021-10-06

**Authors:** Adriana I. Muñoz, Luis Vallejo-Castillo, Ana Fragozo, Said Vázquez-Leyva, Lenin Pavón, Gilberto Pérez-Sánchez, Rodolfo Soria-Castro, Gabriela Mellado-Sánchez, Laura Cobos-Marin, Sonia Mayra Pérez-Tapia

**Affiliations:** 1 Present Address: Departamento de Inmunología. Escuela Nacional de Ciencias Biológicas (ENCB), Instituto Politécnico Nacional (IPN), Prolongación de Carpio y Plan de Ayala S/N, Col. Santo Tomás, Alcaldía Miguel Hidalgo, 11340 CDMX México; 2 Unidad de Desarrollo e Investigación en Bioprocesos (UDIBI), ENCB, Instituto Politécnico Nacional (IPN), Alcaldía Miguel Hidalgo, 11340 CDMX México; 3 Laboratorio Nacional para Servicios Especializados de Investigación, Desarrollo e Innovación (I + D + i) para Farmoquímicos y Biotecnológicos (LANSEIDI-FarBiotec-CONACyT), ENCB, Instituto Politécnico Nacional (IPN), Alcaldía Miguel Hidalgo, 11340 CDMX México; 4 Laboratorio de Psicoinmunología, Dirección de Investigación en Neurociencias, Instituto Nacional de Psiquiatría Ramón de la Fuente, Calzada México-Xochimilco 101, Colonia San Lorenzo Huipulco, Alcaldía Tlalpan, 14370 CDMX México; 5 Laboratorio de Virología, Facultad de Medicina Veterinaria y Zootecnia, Universidad Nacional Autónoma de México, México, 04510 CDMX México

**Keywords:** Immunology, Immunotherapy, Innate immune cells, Neuroimmunology, Translational immunology, Medical research, Translational research

## Abstract

Canine parvovirus type II (CPV-2) infection induces canine parvoviral enteritis (CPE), which in turn promotes sepsis and systemic inflammatory response syndrome (SIRS). Mortality in this disease is usually registered within 48–72 h post-hospitalization, the critical period of the illness. It has been recently described that the use of an immunomodulator, whose major component is monomeric ubiquitin (mUb) without the last two glycine residues (Ub∆GG), in pediatric human patients with sepsis augments survival. It is known that CXCR4 is the cell receptor of extracellular ubiquitin in humans. This work aimed to explore the effect of one immunomodulator (human Dialyzable Leukocyte Extract-hDLE) as a therapeutic auxiliary in puppies with sepsis and SIRS induced by CPE. We studied two groups of puppies with CPV-2 infection confirmed by polymerase chain reaction. The first group received conventional treatment (CT) and vehicle (V), while the second group received CT plus the immunomodulator (I). We assessed both groups' survival, clinical condition, number of erythrocytes, neutrophils, and lymphocytes during the hospitalization period. In addition, hematocrit, hemoglobin, plasma proteins and cortisol values, as well as norepinephrine/epinephrine and serotonin concentration were determined. Puppies treated with CT + I showed 81% survival, mild clinical signs, and a significant decrease in circulating neutrophils and lymphocytes in the critical period of the treatment. In contrast, the CT + V group presented a survival of 42%, severe clinical status, and no improvement of the parameters evaluated in the critical period of the disease. We determined in silico that human Ub∆GG can bind to dog CXCR4. In conclusion, the administration of a human immunomodulator (0.5 mg/day × 5 days) to puppies with CPE under six months of age reduces the severity of clinical signs, increases survival, and modulates inflammatory cell parameters. Further studies are necessary to take full advantage of these clinical findings, which might be mediated by the human Ub∆GG to canine CXCR4 interaction.

## Introduction

The causal agent of canine parvoviral enteritis (CPE) is Canine Parvovirus Type 2 (CPV-2), a lytic virus of the *Parvoviridae* family^1^. CPV-2 is an icosahedral, non-enveloped virus; it has a negative-sense single-stranded genome and a size of 5.5–6.2 kDa^2^. It affects dogs of any breed, sex, and age, but those under six months show higher incidence. The morbidity of the virus is 100%, while mortality among puppies without medical treatment reaches 91%, which can be reduced with veterinary medical care^2,3^.

The infection with CPV-2 has two clinical presentations: viral myocarditis in neonatal dogs of unvaccinated mothers, and CPE with severe gastroenteritis^3^ with mucoid or hemorrhagic diarrhea, vomit, depression, dehydration, prolonged capillary refill time^4^, abdominal pain, hypo- and hyperthermia^2^, besides, under severe conditions, hypovolemic shock^3^. The hematological and biochemical changes that characterize this disease are lymphopenia, neutropenia^5^, hyperazotemia, hypoalbuminemia, metabolic acidosis or alkalosis, and alterations in coagulation^3^.

The treatment of CPE is based on restoring the water balance, correcting metabolic alterations, and preventing secondary bacterial infections^4,6^. Some clinical studies suggest the use of omega interferon^7^, a soluble transferrin receptor (CPV-2 receptor)^8^, antiviral drugs^9,10^, and hyperimmune serum^11^ as treatments aimed at reducing the viral load instead of solving the sepsis and the systemic inflammatory response syndrome (SIRS), critical elements linked to the high mortality rate of CPE^4^.

Physiopathological alterations of CPE, such as secondary bacterial translocation, destruction of intestinal crypts, neutropenia, and immunosuppression due to thymus atrophy, cause sepsis and SIRS in puppies^3,12^. These exacerbate clinical signs and lead to multiple organ dysfunction syndrome and death^12^. There is a high incidence of sepsis and SIRS in puppies with CPE, which makes it a sepsis model for humans^13^.

There have been two recent reports that are fundamental to the present work. The first one is about the increased survival of pediatric human patients with sepsis, treated with the conventional treatment and human Dialyzable Leukocyte Extract (hDLE), an immunomodulator^14^. hDLE reduced serum concentration of C-reactive protein and circulating neutrophils 72 h post-admission and increased lymphocyte count^14^. And the second one is about the main active ingredient in this immunomodulator: the extracellular monomeric human ubiquitin (mUb) and a variant with the two C-terminal glycine residues deleted (Ub∆GG)^15^. It has been reported that extracellular mUb controls inflammation in sepsis, favoring anti-inflammatory activity modulated by the vagus nerve^16,17^.

This work aimed to explore the effect of one immunomodulator as a therapeutic auxiliary in CPE puppies with sepsis and SIRS. So that, we evaluated the clinical symptoms of the disease, survival, numerical changes in neutrophils and lymphocytes counts. Additionally, to assess the vagus nerve tone activity, we determined the plasmatic concentrations of cortisol and the neurotransmitters norepinephrine, epinephrine, and serotonin. To support evidence to the potential mechanism, the second objective of this work was to analyze the in silico interaction of mUb and Ub∆GG with dog CXCR4.

## Results

### Description of the study population

The age, sex, breed, and vaccination status of all participants and the treatment received are described in Table 1. The age (months) of the puppies in the Conventional treatment (CT) + Vehicle (V) group was 3.1 ± 0.9, and that of subjects in CT + Immunomodulator (I) was 3.7 ± 1.1. The statistical analysis showed no age differences between groups. The study population consisted of 45% mixed-breed and 55% pure-breed puppies. Only 4/18 of the puppies showed vaccination history (attenuated, live virus) at two months of age in the sample analyzed. The drugs administered as CT to the CPV-2-infected puppies are listed in Table 2.

**Table 1:** Demographic characteristics of the study population.

Group	Puppy number	Age (months)	Sex	Breed	Vaccination status
CT + V	1	4	Male	Mixed	No
	2	2	Male	Pit Bull	No
	3	2	Male	Chihuahua	No
	4	4	Male	Blood Hound	No
	5	4	Male	Schnauzer	No
	6	3	Male	Mixed	No
	7	3	Male	Poodle	Yes
CT + I	1	3	Female	Mixed	No
	2	2	Female	Mixed	No
	3	4	Male	Mixed	Yes
	4	2	Female	Pomeranian	No
	5	5	Male	Schnauzer	No
	6	4	Female	Pit Bull	No
	7	3	Female	Mixed	No
	8	5	Male	Mixed	No
	9	4	Female	Schnauzer	Yes
	10	4	Male	Schnauzer	Yes
	11	5	Male	Mixed	No

The number of puppies per group, age, sex, breed, and vaccination status is described.

*CT* conventional treatment, *I* immunomodulatory, *V* vehicle.

**Table 2:** Conventional treatment in CT + V and CT + I groups.

Treatment classification	CT + V		CT + I	
Fluid therapy	0.9% NaCl solution	7/7	0.9% NaCl solution	11/11
Antibiotics			Ampicilin	5/11
	Ampicilin	3/7	Metronidazole	8/11
	Metronidazole	5/7	Enrofloxacin	2/11
	Enrofloxacin	2/7	Clindamicyn	1/11
	Cephalexin	5/7	Sulfa-trimethoprim	4/11
Antiemetics	Maropitant citatre	7/7	Maropitant citrate	9/11
	Metoclopramide	4/7	Metoclopramide	5/11
Antiespasmodics	Prifinial	1/7	Butylhioscine	5/11
Gastric mucosa protectors			Ranitidine	8/11
	Ranitidine	4/7	Omeprazole	9/11
	Omeprazole	7/7	Sucralfate	4/11
Analgesics			Buprenorphine	10/11
	Buprenorphine	7/7	Lidocaine	5/11
	Meloxicam	1/7	Metamizole	3/11
Others	0.5% Dextrose	5/7	0.5% Dextrose	8/11
	Aminolyte	2/7		

Treatments were classified according to their action mechanism. The number of puppies that received each of the treatments is specified.

*CT* conventional treatment, *I* immunomodulatory, *V* vehicle.

### Confirmation of CPV-2 infection

All puppies included in this study were confirmed as positive to CPV-2 infection by end-point polymerase chain reaction (PCR) (see Fig. 1 of supplementary information) if the corresponding region (466-bp) was amplified.

### Survival, clinimetric evaluation, weight, and days of hospitalization

**Figure 1:**
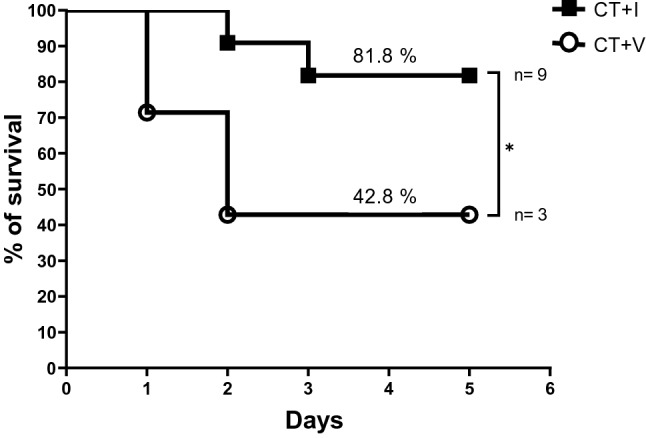
Effect of the immunomodulator on the survival of puppies affected by CPE. Puppies were daily administered with 0.5 mg of the immunomodulator showed an increase in survival (%) vs CT + V group. The initial number of puppies in the CT + V group was 7, and 4 died. The initial number of puppies in the CT + I group was 11, and only 2 died. The difference in survival percentage was 39.0%. Kaplan–Meier test and Gehan-Breslow-Wilcoxon post-hoc test were carried out. *P ≤ 0.05. *CT* conventional treatment, *I* immunomodulatory, *V* vehicle.

The survival results of CT + V *vs* CT + I are represented in Fig. 1, showing a significant increment of the survival rate in puppies co-administered with the immunomodulator (P ≤ 0.043). In addition, it is observed that the deaths in group CT + V occur on day 1 and mainly on day 2, which was defined as the critical day. On the other hand, deaths in the CT + I group occurred on days 2 and 3. Taking the above into account, it is suggested that one or some of the components of the immunomodulator are responsible for the augment of puppies' survival.

**Figure 2:**
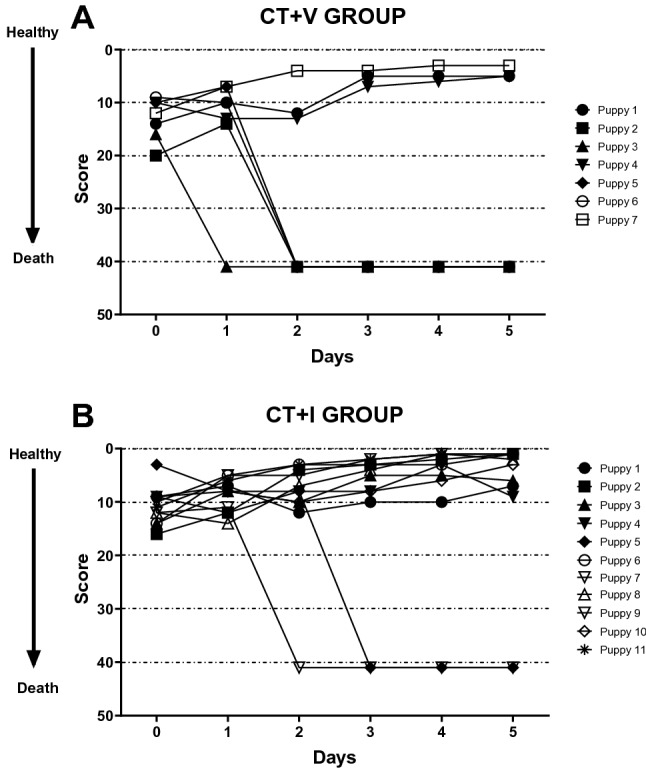
Effect of the immunomodulator over clinical scores of puppies affected by CPE. Clinical score of CT + V (**A**) and CT + I (**B**) across time. Deaths occurred on days 1 and 2 in the CT + V group and days 2 and 3 in CT + I; then, the score of the puppies improved to < 10 in both groups. Each symbol represents a puppy. The score evaluation assigns 0 to the healthy puppy, 21 to high severity state according to Table S1, and, if death occurred, a 20 value was added to accomplish a 41-value score. Kruskal–Wallis test was performed. *CT* conventional treatment, *I* immunomodulatory, *V* vehicle.

The clinical evaluation aimed to identify if the immunomodulator could reduce the severity of the clinical condition in puppies with CPE. Figure 2 represents the clinical score over the time per puppy and group. Regarding the clinical follow-up of the study groups, a high score indicated that the clinical signs were more severe, while a low score meant the severity of the disease was less intense. The arrow points that high values (score from 1 to 41) correlate to a worsening of the clinical condition of the puppy. In all the cases, the score of puppies that died was 41, i.e., 21, the worst clinical condition, plus 20 points if death occurred. The statistical comparison between treatments showed significant differences (*H* = 19.19, *dʄ* = *90,1*; P ≤ 0.024). The CT + I group (Fig. 2B) showed less severe clinical signs than CT + V puppies (Fig. 2A). The improvement in the clinical score of CT + I was observed since the first day of hospitalization and continued in later days, showing values under basal measurement. The CT + V showed an increase in mortality since the hospital admission on day 2; then, the survivor puppies improved the clinical score below the initial value (> 10). Later, we analyzed the data obtained from the survivors of each group on days 1 and 2 and detected no significant differences (*H* = 3.827, *dʄ* = *30,1*; P ≥ 0.280), although, it is observed a tendency of the surviving CT + I puppies to lower scores (healthier) than CT + V group. The rest of the scores were 8.55 ± 3.32 on day 1 and 6.89 ± 3.33 on day 2 in the group CT + I, whereas in CT + V were 10. 00 ± 2.68 on day 1 and 9.67 ± 7.41 on day 2.

Deaths occurred on days 1 and 2 in the CT + V group and on days 2 and 3 in CT + I; afterwards, the score of the puppies improved to < 10 in both groups. This result suggests that the treatment plus the immunomodulator induced a clinical improvement, which was reflected in a lesser mortality rate compared with CT + V. The CT + I puppies that survived the critical period had a better clinical condition. The number of hospitalization days of the surviving puppies was 6.10 ± 2.26 and 7.00 ± 1.00 in CT + I and CT + V groups, respectively; there was no statistical difference.

We did not detect significant differences in the weight between CT + V and CT + I groups (see Figure S2 of supplementary information). However, CT + I puppies showed a positive percentage of weight change at all evaluated times, conversely to the CT + V puppies that showed a negative rate of weight change. For instance, on the critical day of the disease (day 2), the percentage of weight change in the CT + I group was 1.22 ± 4.90, while the CT + V group was − 3.06 ± 5.50. At the end of the study, the CT + I group showed a percentage of 1.85 ± 13.61, and the CT + V group was − 7.72 ± 9.48. A negative change in weight indicates the puppies lost weight with respect to their initial value. In contrast, a positive value is interpreted as weight gain. In this sense, the percentage values ​​of weight change show that the infection with CPV-2 induces weight loss, but the immunomodulator administration hampers it.

### Hematological parameters

Hematocrit, hemoglobin, and total erythrocytes values showed no significant differences at the beginning of the treatments (see Table S2 of supplementary information). The comparison between treatments along the follow-up showed significant differences in hemoglobin levels (*H* = 18.18, *d*ʄ = *82, 1*; P ≤ 0.030); CT + V showed a concentration (g/L) of 104.40 ± 16.83, lower than that of CT + I (127.90 ± 14.81) on day 1. No differences were found in hematocrit, and total erythrocytes count.

Overall, hematocrit, hemoglobin, and total erythrocytes values were higher in CT + I than in CT + V. Along with the clinical follow-up, the mean values of hematocrit (L/L), hemoglobin (g/L), and total erythrocytes (1 × 10^12^/L) in the CT + I group were 0.34 ± 0.06, 113.90 ± 25.40 and 5.43 ± 1.16, respectively; while in the CT + V group were 0.31 ± 0.06, 102.80 ± 18.70, and 5.07 ± 0.93.

The concentration of plasma proteins showed no significant differences between the evaluated treatments (see Table S2 of supplementary information). On the first three days, its values were similar between groups (CT + I = 45.73 ± 10.33 g/L *vs* CT + V = 44.36 ± 9.13 g/L). However, on days 4 and 5, considered recovery days, CT + I showed higher concentrations (day 4 = 44.67 ± 11.43 g/L; day 5 = 47.89 ± 14.05 g/L) than CT + V (day 4 = 35.33 ± 2.52 g/L; day 5 = 36.33 ± 4.62 g/L).

**Figure 3:**
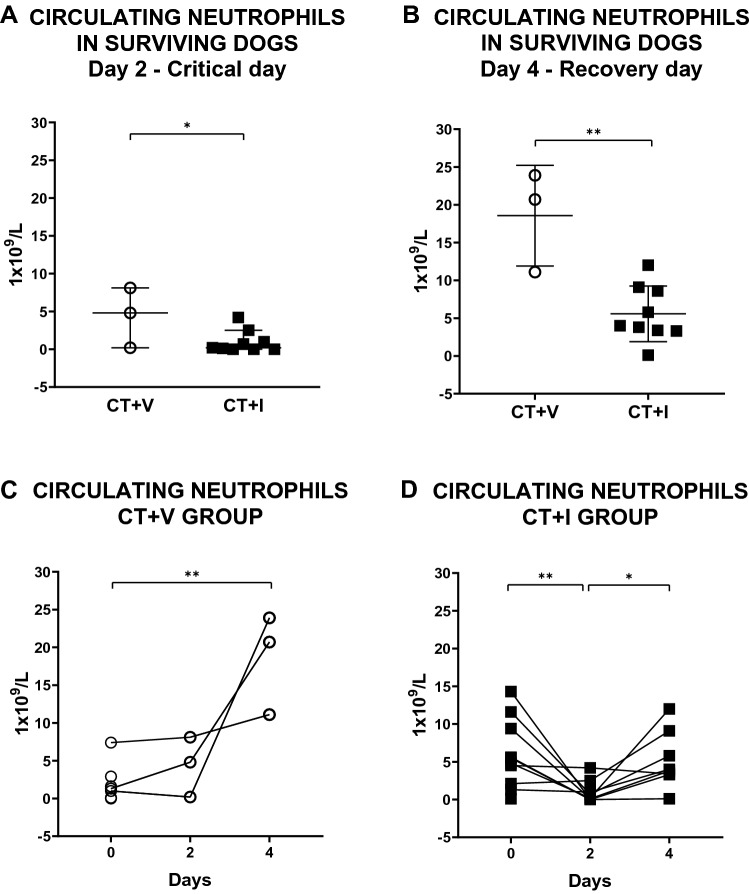
Effect of the immunomodulator over circulating neutrophil concentration of puppies affected by CPE. (**A**) Circulating neutrophils in surviving dogs (day 2-critical day) and (**B**) circulating neutrophils in surviving dogs (day 4-recovery day), statistical differences on days 2 and 4 are due to lower neutrophil concentrations in CT + I than in CT + V. Mann–Whitney test was done. Each dot represents a puppy. Median ± 5–95% confidence interval. (**C**) Neutrophil concentrations on day 0, critical day (2) and recovery day (4) in the CT + V group. Neutrophil concentrations on day 4 are responsible for statistical differences. (**D**) Circulating neutrophils on day 0, critical day (2) and recovery day (4), reduction in neutrophils on day two results in statistical differences in CT + I. Each dot represents a puppy. Kruskal–Wallis test and Dunn's post-hoc test were carried out. *P ≤ 0.05, **P ≤ 0.01. *CT* conventional treatment, *I* immunomodulatory, *V* vehicle.

Regarding the influence of the immunomodulator over the circulating neutrophils concentration, no statistical differences were identified between groups at the beginning of the study (data not shown), but this changed during the study. Firstly, we compared the neutrophils count on days 2 and 4 between groups; the group CT + I exhibited a neutrophil count significantly lower than the CT + V group (Fig. 3A,B). Then, we compared the neutrophil count on days 0, 2 and 4 in an intra-group way. CT + V showed a significantly higher value of neutrophil counts on day 4 when compared to day 0. In addition, although there were no statistical differences, it must be noted that the neutrophil count was lower on day 2 *vs* day 4 (Fig. 3C). On the other hand, a decrement in the neutrophil count was observed in CT + I on the critical day (day 2) concerning days 0 and 4 (Fig. 3D).

**Figure 4:**
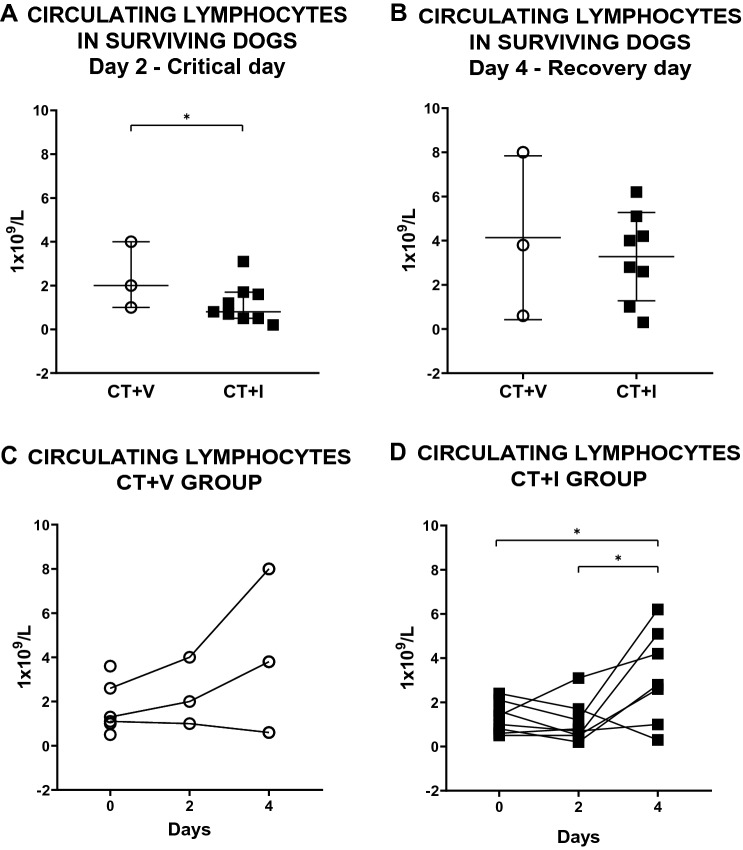
Effect of the immunomodulator over the concentration of circulating lymphocytes of puppies affected by CPE. (**A**) Circulating lymphocytes in surviving dogs on a critical day (day 2) and (**B**) circulating lymphocytes in surviving dogs on recovery day (day 4); there are no statistical differences between groups on day 4; the difference on day 2 is due to the lower lymphocyte values in CT + I *vs* CT + V. Mann–Whitney test was performed. Each dot represents a puppy. Median ± 5–95% confidence interval. (**C**) Lymphocyte concentrations on day 0, critical day (2), and recovery day (4) in the CT + V group. Lymphocyte concentrations do not show statistical differences at any time evaluated. Kruskal–Wallis test and Dunn's post-hoc test were done. Each dot represents a puppy. (**D**) Circulating lymphocytes in CT + I group, this group showed differences in recovery time due to an increase in lymphocytes as compared with basal measurement and day 2. One-way ANOVA, Tukey's post-hoc test. Each dot represents a puppy. Mean ± SD. *P ≤ 0.05. *CT* conventional treatment, *I* immunomodulatory, *V* vehicle.

At the beginning of the clinical follow-up, no differences were detected in the number of lymphocytes between groups (data not shown). Lymphocytes were compared between treatments on the critical day (day 2) and showed significant differences (P ≤ 0.045) (Fig. 4A); however, no differences were found on the recovery day (day 4). On day 2, CT + I exhibited a lower count than CT + V (Fig. 4A,B). When we compared days 0, 2, and 4 per treatment, the CT + I group presented a significant increase in lymphocytes in the recovery period *vs* days 0 and 2 (Fig. 4D). Although CT + V showed no significant differences (Fig. 4C), there was an increase in lymphocytes on day 4.

### Hormone parameters

Statistical analysis did not show differences in cortisol levels between the CT + V and CT + I groups. Despite this, a tendency to decrease cortisol levels is observed in both groups from day 1. It should be noted that the surviving pups of both groups on days 4 and 5 show similar cortisol levels among them (see Figure S3 of supplementary information).

### Neurochemical parameters

**Figure 5:**
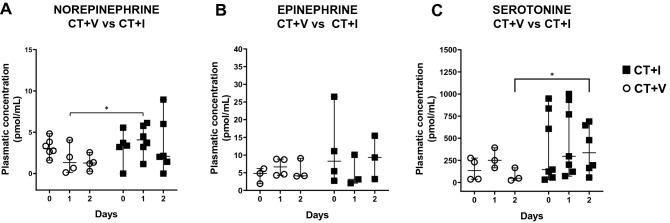
Effect of the immunomodulator over the plasma neurotransmitter concentration of puppies affected by CPE. (**A**) Norepinephrine: A significant increase was observed in the CT + I group on day 1 of evaluation with respect to the CT + V group. (**B**) Epinephrine: No significant differences were observed between groups. (**C**) Serotonin: A considerable decrease was found in CT + V as compared *to* CT + I on day 2. Kruskal–Wallis test and Dunn's post-hoc test were carried out. Each dot represents a puppy. Median ± 5–95% confidence interval. *P ≤ 0.05. *CT* conventional treatment, *I* immunomodulatory, *V* vehicle.

Plasmatic concentrations of neurotransmitters were evaluated on days 0, 1, and 2, considered the critical period of the disease. The comparison of plasmatic norepinephrine levels between treatments showed a significant difference on day 1 (Fig. 5A). It is observed that norepinephrine concentrations are higher in the CT + I group than in CT + V in the evaluated period. No differences were detected in plasmatic epinephrine (Fig. 5B). The CT + I group showed epinephrine values ​​lower on day 1 than the initial value. The serotonin values presented in Fig. 5C evidenced a significant increase only in CT + I on day 2 of follow-up (*H* = 12.93, *d*ʄ = *60,1*; P ≤ 0.035).

### In silico analysis of dog CXCR4 interacting with human mUb and Ub∆GG

**Figure 6:**
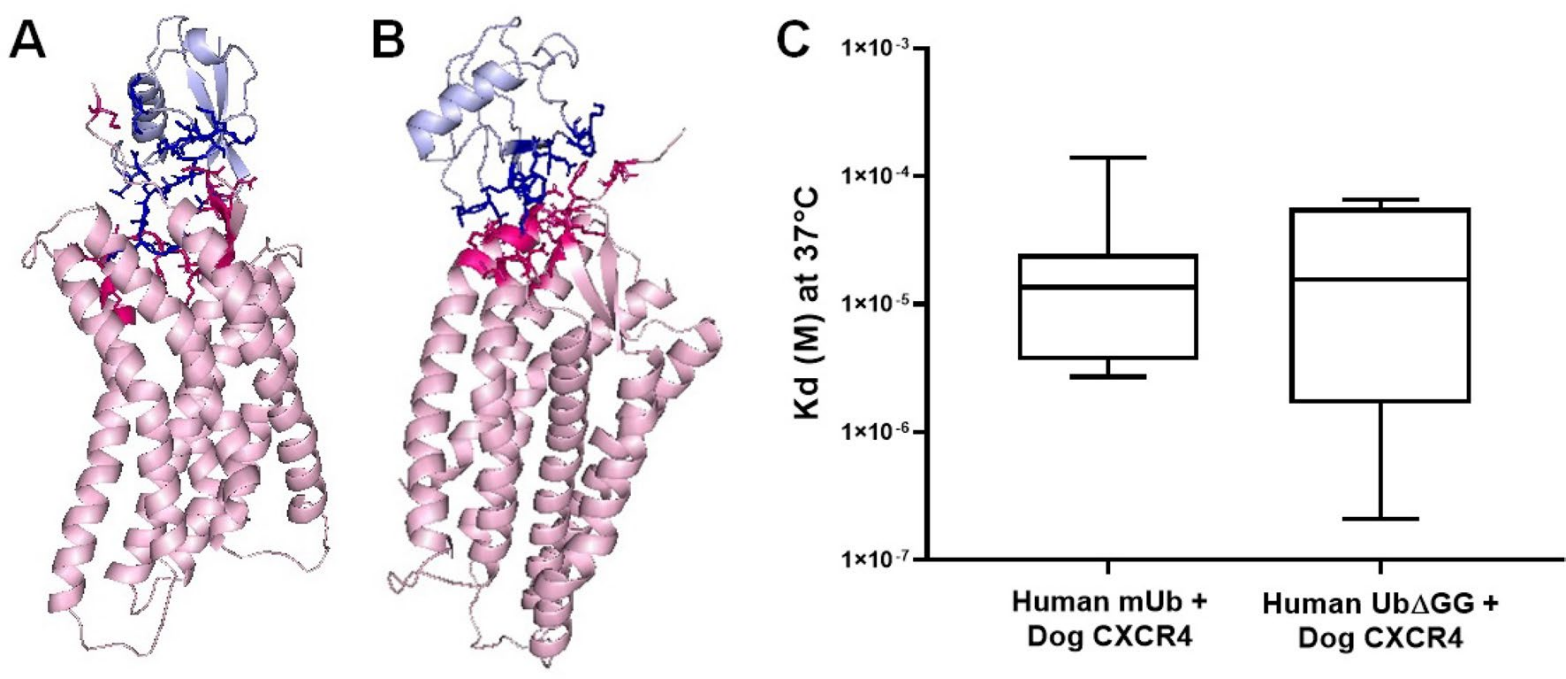
In silico analysis of binding of dog CXCR4 with mUb or Ub∆GG. The coupling analysis of dog CXCR4 with mUb (**A**) or Ub∆GG (**B**) was carried out on the ClusPro 2.0 server. Dog CXCR4 is shown in pink and mUb or Ub∆GG in blue. Dark pink or dark blue indicate the interface area between dog CXCR4 with mUb or Ub∆GG, respectively. The coupling visualization was done with PyMOL v2.0 (**C**), and the value of the dissociation constant (Kd) at 37 °C was assessed from the data obtained with ClusPro and the PRODIGY server. No statistical differences were found between the molecular coupling between the dog CXCR4 with any version of ubiquitin. The graph shows the median + interquartile range obtained in the statistical analysis using the Mann–Whitney test.

The major components of the human immunomodulator used in this study are mUb and Ub∆GG, for which endogenous receptor in human is CXCR4^18^. To predict if mUb and Ub∆GG might elicit an effect in dogs through the orthologous receptor, we performed a docking assay (Fig. 6A,B). The theoretical values of the constant dissociation (Kd) of mUb–CXCR4 and Ub∆GG–CXCR4 were 1.35 x 10-5 and 1.55 x 10^-5^, respectively (Fig. 6C), which were statistically similar (Fig. 6C), which means that, from a biological standpoint, that the major components of the human immunomodulator could indistinctively bind to dog CXCR4. At the same time, the binding affinity is not affected by the loss of both glycines.

Interestingly, we observed that the Kd at 37 °C between dog CXCR4 with human mUb was 13.5 nM; this value is much lower than the Kd at 37 °C reported for the binding of human CXCR4 to human mUb (84 nM) by experimental assays^18^. However, this could be due to the inherent bias of our in silico assay compared to experimental data previously reported. On the other hand, we evaluated the in silico interaction of human CXCR4 with human mUb, finding that the Kd at 37 °C was 0.176 nM (data not shown), a much lower value than the interaction of dog CXCR4 with human mUb. These results suggest that the binding affinity of human mUb to human CXCR4 is much higher than that of dog CXCR4.

## Discussion

CPE is the most common and severe gastrointestinal disease among puppies aged six months or less^3^. The development of sepsis during CPE infection is frequent and secondary to physiopathological alterations of this disease, i.e. bacterial translocation and lymphopenia^3,4^. In addition to the gastrointestinal symptoms of CPE, it is common to detect hematological alterations in CPV-2 infected puppies, which are characterized by a reduction of red blood cells and leukocytes, such as neutrophils and lymphocytes^4^. These events may be associated with thymus atrophy, gastroenterological bleeding losses, hyper inflammation, and CPV-2 tropism towards the bone marrow precursors^1,5^. All these physiological alterations generate a high mortality rate^4^.

In this study, our group has demonstrated that administering a human immunomodulator as an auxiliary treatment for puppies affected by CPE increases survival. The CT + I group showed an increase of ~ 40% of survival in comparison with conventional treatment. In other cases where immunomodulators have been used, the researchers have reported similar effects in pediatric human patients with sepsis^14^ and likewise in a murine model of Herpes virus type 1 infection^19^. We chose post-hospital day 2 as the critical period for this study because the highest percentage of mortality (4/6) was observed in the CT + V group. This information agrees with previous reports, where the mortality in CPE puppies is observed within 48 h after the appearance of clinical signs^2^. This period's importance is that most prognostic biomarkers are defined between 24 to 48 h after hospitalization^20,21^. Mortality on day 1 can be explained by the presence of severe sepsis, metabolic complications or comorbid conditions^4^, while puppies that died on the third day are in the period in which mortality often occurs^22^.

Considering that there is no international consensus treatment for parvovirus infection, each veterinarian was allowed to apply the required pharmacologic treatment to the puppies according to their criteria. Nevertheless, it is worth noting that the type of drugs used was similar among the experimental groups. In the case of antiemetics, there is evidence that metoclopramide and maropitant have similar effects in CPV-2 infected puppies^23^. In addition, it is reported that puppies with CPV-2 have the same outcome in standard in-hospital and outpatient treatments^24^. In this sense, the positive effects in CT + I were merely associated with the addition of the immunomodulator instead of the pharmacological treatment or the clinic where the puppies were treated.

In terms of the clinical scores, our results show that the CT + I group presented better clinical conditions in each of the variables evaluated compared to the CT + V group (data not showed). The clinical improvement may be related to administering the immunomodulator, which favored better hematocrit, hemoglobin, and erythrocyte and plasma protein concentrations on days 4 and 5 compared to the CT + V group. These findings suggest that the CT + V group suffered more significant damage to the intestinal epithelium, resulting in greater loss of enteric proteins at days 4 and 5 and reduced red blood cell count. Other findings that support this are the worse clinical score and weight loss in the CT + V group, variables that showed a better outcome in the CT + I group.

Cortisol is one of the most often used endocrine biomarkers in CPE management^25,26^. Levels greater than 224 mmol/L at 48 h post-admission negatively affect puppy survival^21^; the cortisol cut-off is not a general rule, as observed in our study groups. It is essential to consider that the hyperactivity of the hypothalamic–pituitary–adrenal axis, which is active in infectious diseases, increases circulating levels of cortisol^27^. At prolonged high cortisol concentrations, this hormone can generate immunosuppression and consequently increase mortality^28^. In our work, cortisol concentration tends to decrease in the surviving puppies in both groups, which reduces the risk of mortality.

Blood cell count has been used as a biomarker of severity and prognosis due to its low cost and accessibility since the first clinicopathological descriptions of CPE^29^. Our results show that the CT + I group has lower circulating neutrophils and lymphocytes on day 2 (critical day) and for neutrophils only on day 4 of hospitalization (recovery period) than the CT + V group. We proposed that the decrease in circulating neutrophils and lymphocytes in the CT + I group is due to vagal anti-inflammatory arc-mediated immunomodulatory activity. The indirect evidence of immunomodulation is present in the CT + I group if we consider the predictive values according to the neutrophil and lymphocyte counts in the critical phase of the disease that suggested a high mortality rate. However, despite them, the CT + I group presents an increase in survival compared to the CT + V group.

There are two pathways of activation of the vagus nerve to regulate the inflammatory response: the vagal cholinergic and the splenic sympathetic pathway (SSP)^30^. The vagal cholinergic pathway is regulated by efferent vagal fibers, which synapse with neurons of vagus nerve branches widely distributed in the organism tending to release acetylcholine (ACh) at the synaptic cleft. ACh can bind to the α7-nicotinic receptor (α7-nAChR) of macrophages to transcriptionally inhibit the production of proinflammatory cytokines as TNF-α, IL-1β, and IL-6^30,31^. In the SSP, the vagus nerve stimulates the splenic sympathetic nerve releasing norepinephrine through the β2-adrenergic receptor; norepinephrine promotes ACh production by an overpopulation of splenic CD4^+^ memory T lymphocytes (CD4^+^CD44^high^CD62L^low^)^32^. These lymphocytes express acetylcholine transferase, the enzyme that catalyzes ACh production from choline and acetyl-CoA^33^. The vagus nerve activation effectively reduces the severity of many inflammatory disease models, including endotoxemia^34,35^ and colitis^36^.

Additionally, the cholinergic effect is not limited to the inflammatory response. For instance, the increase of ACh levels favors the decrease of circulatory levels of hormones and neurotransmitters, such as cortisol and epinephrine, respectively^37^. In our study, the changes in plasmatic levels of cortisol and neurotransmitters observed since day 1 in the surviving puppies also suggest that the immunomodulator administration increases the cholinergic tone.

The main components of the immunomodulator used in this work have recently been published: mUb and Ub∆GG^15^. Highly conserved among mammals^16^, the extracellular mUb has a low molecular weight and controls inflammation in sepsis through activation of CXCR4^17^. The anti-inflammatory effect of ubiquitin may be due to direct action with the CXCR4 receptor expressed in macrophages and neutrophils and the vagus nerve^15,38^; this interaction was indirectly explored in our work. The literature suggests that a systemic effect modulated by the vagal anti-inflammatory arc should generate a decrease in soluble and cellular inflammatory parameters (lymphocyte and neutrophil counts), a reduction of plasmatic levels of epinephrine and cortisol^37^. Based on these facts, we propose that the participation of the vagal anti-inflammatory arc is one of the mechanisms activated by ubiquitin that improves the clinical, cellular, and neurochemical aspects.

The mUb and Ub∆GG may act in two possible ways. As previously described, the first one involves the interaction with CXCR4 receptors in macrophages and neutrophils, inducing a decrease of proinflammatory cytokine release, the respiratory burst, and the migratory capacity of these cells^17,38^. On the other hand, mUb and Ub∆GG may interact with CXCR4 receptors expressed on the vagal endings at the site of immunomodulator administration^15^. In vitro studies have demonstrated that β2-adrenergic receptors negatively modulate the neutrophil oxidative burst, chemotaxis, the formation of neutrophil extracellular traps, and the expression of adhesion molecules leukotriene B4, chemokines, and cytokines^39,40^. The effects of ACh on neutrophils have been investigated using nicotine as an α7-nAChR agonist. It has been demonstrated that activating nicotinic receptors can inhibit the massive recruitment of neutrophils in vital organs, preserving tissue integrity^41^. Besides, the activation of nicotinic receptors in lymphocytes produces a reduction in their proliferation and differentiation^42^, and it has been observed in a rat model of sepsis that 7-α-nAChR activation may improve the functional indices of activated lymphocytes and protect against lethality^43^. Our data are insufficient to identify which of these mechanisms are present in puppies due to the diversity and complexity of the biological effects mediated by β-adrenergic receptor activation on lymphocytes. This phenomenon depends on factors such as the time of receptor activation, the activation state of the lymphocyte, the subset of lymphocyte, the signaling pathway involved, and the cytokine microenvironment^44^. Future studies must be performed to validate the participation of the inflammatory vagal arc in the increase of survival in puppies with CPE, whose main objective will be the evaluation of the nicotinic receptors.

Another finding related to the determination of neurotransmitters was the statistical difference in norepinephrine observed on day 1 and serotonin concentrations observed on day 2. The CT + I group showed increased concentrations of norepinephrine and serotonin than the CT + V group. The increase in both plasmatic neurotransmitters in the puppies that received the immunomodulator could correlate with the improvement in mental state^45,46^; most of the puppies were less depressed (data not shown).

Moreover, at the time of writing, there is no report on the interaction between dog CXCR4 and human mUb. One reason could be that dog CXCR4 has not yet been crystallized, so we had to model it as indicated in the corresponding section. Although the binding affinity of human CXCR4 to human Ub is higher than that observed for dog CXCR4, which was expected, this interaction could be sufficient to generate a biological effect such as augmenting survival in puppies administered with the immunomodulator.

To the best of our knowledge, this is the first report about the interspecies effect of this human immunomodulator in dogs and in silico analysis that was showing the possible molecular interaction between mUb or Ub∆GG with dog CXCR4. As suggested by the in silico molecular docking results, the positive effects observed in this work are likely due to human mUb/Ub∆GG and dog CXCR4 triggering the suggested vagal modulation mechanisms (vide supra).

Finally, even though sex hormones are involved, it should be noted that there is no predisposition due to sex to develop CPE among dogs younger than 6 months^3^. On the contrary, all breeds are susceptible to CPE, Rottweilers, Doberman Pinschers, American Pit Bull Terriers, Labrador Retrievers, English Springer Spaniels, and German Shepherds are at a higher risk^1, 13^. It has been documented that mixed-breed dogs are less susceptible to CPE than their pure breed counterparts^3^, while other researchers have found no breed predisposition^47^.

Study limitations: To adequately benefit from the results obtained in this work, we must state that among the limitations is the number of recruited animals, mostly CT + V. The puppies were multicentrically recruited and randomly assigned to the experimental groups. Those that did not comply with the inclusion criteria were removed. It induced that the size of the groups was different along with the high mortality rate of the disease, promoted that the effect of sample size was important. Therefore, future studies should consider a larger control group so that the number of surviving participants allows more efficient statistical comparisons. In addition, further studies will consider strategies to measure soluble inflammatory parameters as cytokines because, in the present work, the volumes drawn were small, and clinimetric measurements were privileged for a pilot study to support a more extensive study.

## Conclusions

Our result suggests that using an immunomodulator in CPE infection increases puppy survival and improves clinical conditions because it promotes decreased neutrophils and lymphocyte counts at the critical stage of the disease, reducing the cortisol and neurotransmitter changes plasmatic levels. These encouraging findings indicate the efficacy of the immunomodulator as a therapeutic aid and could consolidate its use as a treatment option in CPE. However, further research will be necessary to take advantage of the findings obtained from this first experimental approach.

## Materials and methods

### Study population

Eighteen puppies with CPE from five veterinary hospitals in the metropolitan area of the Valley of Mexico were included between March and September 2019. All the puppies met the inclusion criteria of the research protocol (FTU/P3/19/03) as approved by the Ethics in Research Committee of Escuela Nacional de Ciencias Biológicas at Instituto Politécnico Nacional (ENCB-IPN), equivalent to IACUC regarding Handling and Care of Animals. All the tutors signed informed consent letters. The manuscript follows the recommendations in the ARRIVE guidelines, and all experiments were performed in accordance with relevant guidelines and regulations.

The inclusion criteria of this study can be consulted in Table S3 of supplementary information. We included CPE puppies under 6 months of age, regardless of sex and breed, that exhibited at least two clinical criteria of SIRS secondary to sepsis (hypothermia < 37.8 °C, fever > 39.4 °C, tachycardia > 140 bpm, tachypnea > 30 breaths/min, leukopenia < 5.5 × 10^9^/L, and leukocytosis > 12.5 × 10^9^/L)^7^. Puppies were randomly assigned to any of the two study groups. The group with CT + V, (*n* = 7) or the group (CT + I, *n* = 11). Once the puppies were approved for the study, they were admitted and remained under the care of veterinary doctors at the participant center. Each veterinarian was allowed to apply the required pharmacologic treatment to the puppies according to his criteria.

### Immunomodulator

The immunomodulator used is an hDLE, commercialized as Transferon® (batch 18A03), kindly donated for this protocol by Pharma-FT at ENCB-IPN (Mexico City, Mexico)^48^. Briefly, the leukocyte-platelet concentrates of healthy human donors were collected at blood banks in Mexico City and lysed through freezing/thawing cycles. After lysis, the active pharmaceutical ingredient (API) was obtained with tangential flow filtration, using a 10-kDa molecular weight cut-off cassette. The hDLE was expressly formulated for this study at 0.5 mg/mL of total protein concentration using UV absorbance at 280 nm. The final product passed the identity test by molecular size chromatography, endotoxin content by *Limulus* amebocyte lysate, and microbial limits according to the Pharmacopeia of the United Mexican States^49^. Then, the hDLE was stored in 10 mL sterile glass bottles with bromobutyl stoppers in laminar flow cabinets and handed over to the head researcher of the study along with the same number of vials containing water for injection (Laboratorios PiSa, Jalisco, Mexico). Both products were stored and transported in refrigeration (5 ± 3 °C) to the administration site.

### Administration of conventional treatment and immunomodulator

Puppies were randomly assigned to two different groups once tutors gave their consent. Both groups were conventionally handled as recorded in the files of each puppy. In addition, the control group (CT + V) received the vehicle, water for injection, and the other group (CT + I) was administered the immunomodulator (0.5 mg in 1 mL vehicle, daily). Treatments were administered subcutaneously in the interscapular region every 24 h for 5 days.

### Sample collection: feces and whole blood

In all the cases, two fecal samples were collected using a sterile swab directly in the rectum. The first one was used as screening using rapid detection of CPV-2 by immunochromatography (Anigen Rapid CPV Ag Test kit, Bionote, South Korea), and the second helped confirm the diagnosis by end-point PCR. The sample was placed in a test tube without anticoagulant (BD Vacutainer^®^, New Jersey, USA) with 1 mL sterile saline solution and kept at − 20 °C. It was then placed in a sterile microcentrifuge tube and centrifuged at 12,600 × *g* for 10 min. We added DNAzol (Invitrogen^®^, USA) to the supernatant obtained (400–600 µL) at a 1:1 ratio and kept it at − 75 °C until the extraction of genetic material.

Blood samples were extracted from the jugular vein at days 0, 1, 2, 3, 4, and 5. The blood was placed in tubes with anticoagulant EDTA (BD Vacutainer®, New Jersey, USA) and kept under refrigeration (5 ± 3 °C) until its analysis. The amount obtained (1–3 mL) was adjusted to the puppy's weight per day, caring for the well-being of the animal. Once the hematological parameters were assessed, samples were centrifuged at 1,233 × *g* for 10 min. The plasma obtained was stored at − 20 °C until cortisol and neurotransmitters were quantified. The samples were obtained between 9 a.m and 12 p.m.

### Confirmation of CPV-2 infection

Infection was confirmed at the Virology Laboratory of the Facultad de Medicina Veterinaria y Zootecnia (FMVZ) at Universidad Nacional Autónoma de México (UNAM). To extract the genetic material, 500 µL DNAzol (Invitrogen®, Massachusetts, USA) and 8 µL Proteinase K (Bioline, London, UK) were added at a concentration of 60 µg/mL to the stored sample (500 µL) at − 75 °C. The mixture was incubated at 56 °C for 30 min, and DNA was extracted according to the DNAzol reagent protocol, using 100 and 75% molecular biology grade ethanol (Sigma Aldrich, Darmstadt, Germany). The precipitate was hydrated with 30 µL DNAse-free water, and DNA was quantified by spectrophotometry in a NanoDrop spectrophotometer. The end-point PCR was carried out following the protocol of Master Mix Platinum II PCR MM (Invitrogen®, Massachusetts, USA) in a MultiGene® Optimax thermal cycler with an initial denaturation temperature of 95 °C for 10 min and subsequently, 30 cycles of denaturation at 95 °C for 30 s, annealing at 55 °C for 30 s, and extension at 72 °C for 30 s; the total time of the PCR was 2 h and 30 min. The primers used forward 5’-GAC CAG CTG AGG TTG GTT ATA G- 3' and reversed 5'- GGT GCA TTT ACA TGA AGT CTT GG 3' were directed to the gene coding for the capsid protein (VP2) of CPV-2 and created a 466-bp amplicon. They were designed by the research group at FMVZ, based on the publication by Balasubramaniam et al.^50^. The electrophoresis was carried out in a 2% agarose gel stained with ethidium bromide for analysis in a UV transilluminator.

### Clinimetric evaluation, weight, survival time, and days of hospitalization

The clinical evaluation took place across 5 days, between 8:00 a.m. and 11:00 a.m., according to the guidelines of the diagnostic methodology to perform a detailed clinical examination. The veterinarians in charge of the puppies received training before the study to homologate clinical evaluation criteria. The parameters evaluated were temperature, mental health (alert and responsive, depressed or in coma), dehydration percentage, degree of abdominal pain, characteristics of feces (pasty stool, mucoid diarrhea, hemorrhagic diarrhea), presence and characteristics of vomit, and death^7^. Each parameter was given a measuring scale; a higher score indicated the severity of the disease was greater. Table S1 of supplementary information specifies the clinical parameters evaluated as well as their scores. Weight was recorded during the clinical inspection.

The survival time record started 24 h after puppies were admitted and ended at day 5. Days of hospitalization were quantified up to hospital discharge, which took place once the puppies' consumer feed and water in the absence of vomit for more than 24 h and showed no clinical signs of depression, fever, or hypothermia.

### Hematological parameter assessment

Blood samples were sent to a private veterinary clinical laboratory for a complete hemogram. Hematocrit, hemoglobin, and total erythrocyte and leukocyte counts were assessed using a CELL-DYN® Emerald hematology analyzer (Abbot, Illinois, USA), which is based on electronic impedance and absorption spectrophotometry. The analyzer was calibrated and verified for the target species. The number of circulating neutrophils and lymphocytes was obtained from a blood smear with a differential leukocyte count. This information was used to obtain the absolute value based on the total number of leukocytes. Plasma proteins were identified in a conventional refractometer (RHC-200act, Mexico).

### Plasmatic cortisol quantification

Plasmatic cortisol was determined in a private veterinary clinical laboratory. The plasma samples were processed in an Architect i2000SR immunoassay analyzer (Abbot, Illinois, USA). The method was based on a heterogeneous chemiluminescent microparticle immunoassay.

### Quantification of plasmatic norepinephrine, epinephrine, and serotonin

Norepinephrine, epinephrine, and serotonin were determined at the Psychoimmunology Laboratory, Instituto Nacional de Psiquiatría Ramón de la Fuente Muñiz based on the analytical method based on Lara-Espinosa et al. report^51^. Briefly, we extracted norepinephrine, epinephrine, and serotonin from 250 µl plasma by adding 250 µl extraction buffer containing 5% ascorbic acid, 200 mM sodium phosphate, 2.5 mM L-cysteine, 2.5 mM EDTA, and 2.4 M perchloric acid. The mixture was incubated at − 20 °C for 10 min. The supernatants containing norepinephrine, epinephrine, and serotonin were collected after centrifugation at 12,419 × *g* and 4 °C for 10 min. They were processed by solid-phase extraction (SPE) using a Hypersep C18 cartridge (Thermo Scientific, Massachusetts, USA) without activation to retain lipids and recover neurotransmitters, and samples were passed through 0.22 µm filters. Neurotransmitter concentrations were identified by reversed-phase HPLC (RP-HPLC) in a system consisting of a PU-2089 plus pump (Jasco Inc., Japan) and an X-LC™ 3120FP fluorescence detector (Jasco Inc., Japan). The instruments were controlled using ChromNav software (Jasco Inc., Japan). Chromatographic runs were carried out with a Jupiter C18 column (300 Å, 5 µ, 4.6 × 250 mm, Phanomenex®) at 30 °C. The column was in equilibrium with mobile phase A containing 0.1% trifluoroacetic acid in water. A linear gradient was run from min 5 to min 20 with mobile phase B containing 0.1% trifluoroacetic acid in acetonitrile. The flow rate was 0.8 mL/min. The fluorescence detector was set at a gain of 1000, attenuation of 32, the response of 20 s, excitation at 280 nm and emission of 315 nm. The injection volume of the sample was 80–100 µL.

### In silico analysis of the interaction between dog CXCR4 and human mUb and Ub∆GG

The crystallized structure of human CXCR4 contained in the Protein Data Bank (PDB; www.rcsb.org) ID. 4RWS was edited using Biovia Discovery Visualizer Studio software (BDVS, Discovery Studio Modeling Environment, 2017; Dassault Systèmes) to eliminate accessory proteins (vMIP2 and T4 lysozyme). In addition, the intramolecular loops of human CXCR4 were recovered using the *Protein Preparation Wizard* tool of Maestro v12.5 (Schrödinger Suite, Protein Preparation Wizard, 2021-1; Schrödinger). Edited human CXCR4 was used to model dog CXCR4 (*Canis lupus familiaris*; NP_00101041491.1) by homology in Modeller v9.25^52^. The model with the lowest DOPE (Discrete Optimized Protein Energy) score was chosen among the five candidate models.

The crystallized structure of 6KDU obtained from PDB was used as a model after edition with BDVS to eliminate accessory molecules (adenine 5-phosphoribose, Zn + 2, and Mg + 2) to carry out the homology modelling of mUb and Ub∆GG. The model with the lowest DOPE score was chosen among the candidates for mUb and Ub∆GG.

To couple dog CXCR4 to human mUb or Ub∆GG, we used a masking template generated on PyMOL v2.0 (The PyMOL Molecular Graphics System version 2.0; Schrödinger) to direct the interaction specifically in the extracellular domain of CXCR4. The coupling was generated using the ClusPro 2.0 server^53^, choosing the interaction mode *Electrostatic-favored*. The 10 major groups obtained were analyzed to identify the dissociation constant at 37 °C with the PRODIGY server (prediction PROtein binding enerGY). The coupling visualization was produced with PyMOL software.

### Statistical analysis

All statistical tests were run on GraphPad Prism v8.0.1 (San Diego, California, USA). In all cases, data were classified by group or evaluated time and were applied a Shapiro–Wilk test to assess normality. Once two groups with normal distribution were compared, one-way ANOVA and Tukey's post hoc test was applied. Alternatively, Kruskal–Wallis and Dunn's post hoc tests were used. Student t and Mann–Whitney tests were applied to compare the age of puppies and period of hospitalization, respectively.

When comparing weight in all the cases, we used the values resulting from the weight change percentage formula (see Eq. 1).

1 $$Weight \, change \, percentaje=\left[\left(\frac{final \, weight-initial \, weight}{initial \, weight}\right)*100\right]$$

Then, Kruskal–Wallis and Dunn's post hoc tests were carried out. The data analysis of the clinical score was using a Kruskal–Wallis test was done. The survival was assessed with a Kaplan–Meier survival curve and a Gehan-Breslow-Wilcoxon post-hoc test. To evaluate the differences induced by the treatments evaluated in circulating neutrophil and lymphocyte counts, we used the values obtained on days 0, 2, and 4. The neurotransmitter comparison was evaluated on days 0, 1, and 2 through Kruskal–Wallis and Dunn's post-hoc tests. In all cases, there was a statistical difference when P ≤ 0.05, and data were described with mean ± SD.

### Ethics approval and consent to participate

This study was approved by the Ethics Committee of ENCB-IPN (FTU/P3/19/03). All tutors of the recruited puppies gave their written consent.

## Supplementary Information

Supplementary Information 1.

Supplementary Information 2.

Supplementary Information 3.

Supplementary Information 4.

Supplementary Information 5.

Supplementary Information 6.

## Data Availability

All the data sets generated and/or analyzed along the study that support our findings are not publicly available since this work is part of the research at UDIBI, ENCB-IPN. However, they are available through the corresponding authors at a reasonable request at any time.

## Abbreviations

CPE: Canine parvoviral enteritis
SIRS: Systemic inflammatory response syndrome
mUb: Monomeric ubiquitin
UbΔGG: Ubiquitin without the last two glycine residues
CXCR4: Chemokine (C-X-C motif) receptor 4
CT: Conventional treatment
V: Vehicle
I: Immunomodulator
CPV-2: Canine parvovirus type 2
hDLE: Human Dialyzable Leukocyte Extract
ACh: Acetylcholine
α7-nAChR: α7-Nicotinic receptor
SSP: Splenic sympathetic pathway
