# Supplementary material for: Increased survival in puppies affected by Canine Parvovirus type II using an immunomodulator as a therapeutic aid
Source: Sci Rep. 2021 Oct 6;11:19864. doi: 10.1038/s41598-021-99357-y (PMC8494837; doi:10.1038/s41598-021-99357-y)
Supplement: Supplementary file 1 — Supplementary Information 1. [file 41598_2021_99357_MOESM1_ESM.pdf]

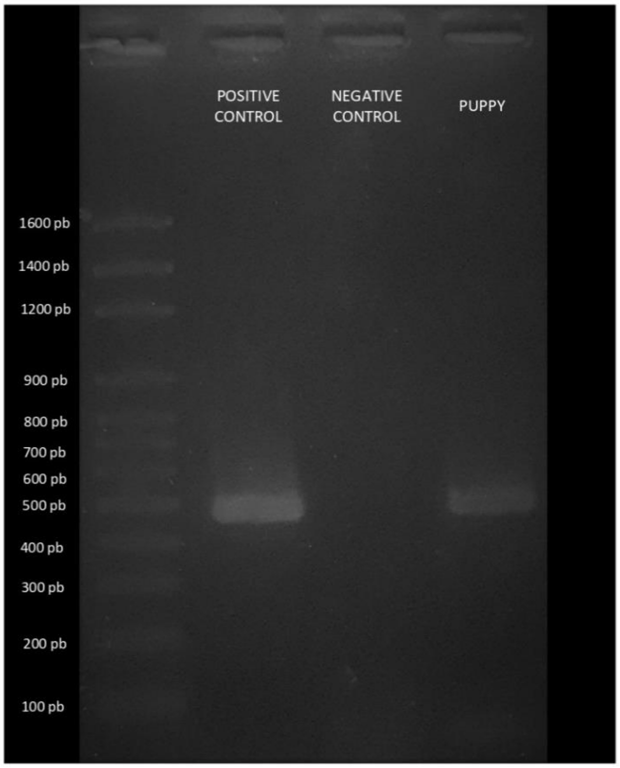

Figure S1. Representative figure of a positive result for CPV-2 infection belonging to a puppy in the CT+I group. A 466-bp amplicon determines a positive result.
