# Supplementary material for: Increased survival in puppies affected by Canine Parvovirus type II using an immunomodulator as a therapeutic aid
Source: Sci Rep. 2021 Oct 6;11:19864. doi: 10.1038/s41598-021-99357-y (PMC8494837; doi:10.1038/s41598-021-99357-y)
Supplement: Supplementary file 2 — Supplementary Information 2. [file 41598_2021_99357_MOESM2_ESM.pdf]

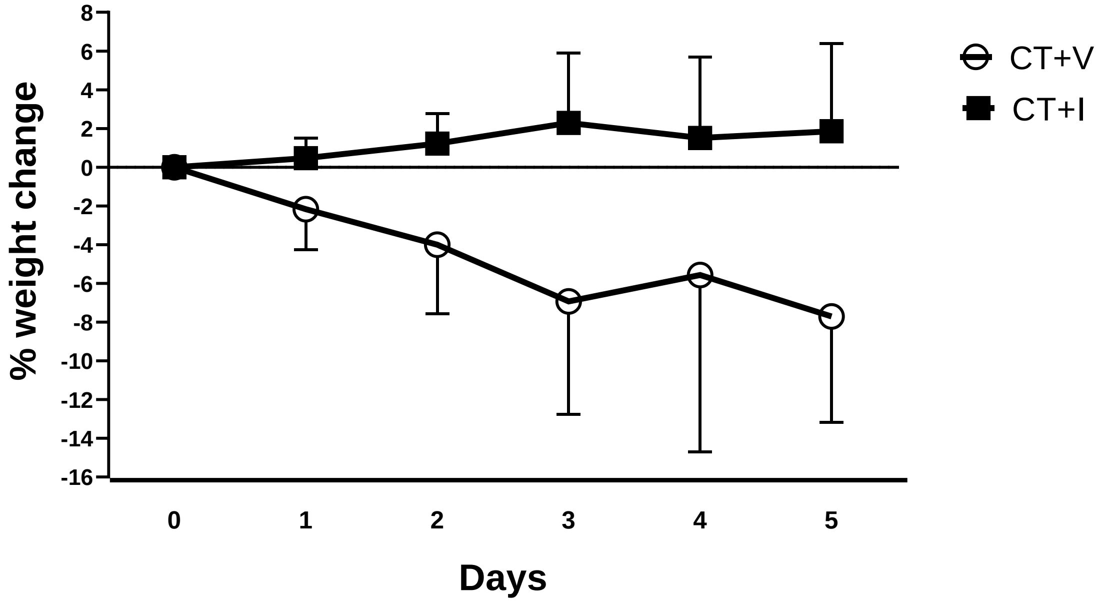

Figure S2. Effect of the immunomodulator over the percentage of weight change of puppies affected by CPE. The CT+I group has a positive weight change percentage from day 1, while the CT+V group is negative. A positive weight change percentage indicates weight gain, and a negative means weight loss. The graph shows the kinetics of the percentage of weight change in the days evaluated. Each symbol represents the mean ± SEM. Kruskall Wallis and Dunn´s post hoc test showed that there were no statistical differences between groups, (H= 10.55, df= *87,1*; $P \geq 0.308$). CT= conventional treatment; I= immunomodulator; V= vehicle.
