# Supplementary material for: Increased survival in puppies affected by Canine Parvovirus type II using an immunomodulator as a therapeutic aid
Source: Sci Rep. 2021 Oct 6;11:19864. doi: 10.1038/s41598-021-99357-y (PMC8494837; doi:10.1038/s41598-021-99357-y)
Supplement: Supplementary file 3 — Supplementary Information 3. [file 41598_2021_99357_MOESM3_ESM.pdf]

Table S1. Evaluated clinical parameters and numerical scale to determine the severity of the disease in puppies with CPE and sepsis.

Parameter	Findings	Score
Rectal temperature	≥ 39.5°C	1
	37.1–37.4°C	2
	≤ 37°C	3
State of mind	Responsive and alert	1
	Depression	3
	Comma	4
Dehydration percentage	5–6%	1
	7–9%	3
	> 10%	4
Feces	Pasty stool	1
	Mucoid diarrhea	2
	Hemorrhagic diarrhea	4
Abdominal pain	Light	1
	Moderate	2
	Severe	3
Vomiting	Vomiting	2
	Hematemesis	3
Death	---	20
