# Supplementary material for: Increased survival in puppies affected by Canine Parvovirus type II using an immunomodulator as a therapeutic aid
Source: Sci Rep. 2021 Oct 6;11:19864. doi: 10.1038/s41598-021-99357-y (PMC8494837; doi:10.1038/s41598-021-99357-y)
Supplement: Supplementary file 4 — Supplementary Information 4. [file 41598_2021_99357_MOESM4_ESM.pdf]

Table S2. Hematocrit, hemoglobin, erythrocytes, and plasma proteins in the puppies infected with CPV-2.

Variable	Group	Day 0	Day 1	Day 2	Day 3	Day 4	Day 5
Hematocrit L/L	CT+V	0.37 ± 0.60	0.31 ±0.05	0.31 ± 0.07	0.32 ± 0.05	0.31 ± 0.06	0.29 ± 0.70
	CT+I	0.40 ± 0.05	0.38 ± 0.05	0.35 ± 0.05	0.35 ± 0.05	0.34 ± 0.07	0.35 ± 0.07
Hemoglobin g/L	CT+V	122.70 ± 19.33	104.40 ± 16.83*	103.30 ± 22.51	107.70 ± 17.58	102.30 ± 19.11	95.33 ± 21.10
	CT+I	133.60 ± 16.13	127.90 ± 14.81*	118.00 ± 15.17	116.90 ± 16.52	113.20 ± 20.90	119.10 ± 23.33
Erythrocytes $1x10^{12}/L$	CT+V	6.06 ± 0.98	5.16 ± 0.90	5.10 ± 1.17	5.33 ± 0.91	5.03 ± 0.83	4.67 ± 1.10
	CT+I	6.47 ± 0.91	6.03 ± 0.77	5.60 ± 0.58	5.60 ± 0.74	5.38 ± 0.95	5.74 ± 1.08
Plasmatic protein g/L	CT+V	51.57 ± 19.38	43.20 ± 9.83	46.67 ± 9.24	44.00 ± 1128	35.33 ± 2.52	36.33 ± 4.62
	CT+I	56.45 ± 7.94	49.73 ± 11.41	44.50 ± 8.98	42.22 ± 9.78	44.67 ± 11.43	47.89 ± 14.05

The data are described with the mean ± SD. * P ≤ 0.05. CT= conventional treatment; I= immunomodulator; V= vehicle
