# Supplementary material for: Increased survival in puppies affected by Canine Parvovirus type II using an immunomodulator as a therapeutic aid
Source: Sci Rep. 2021 Oct 6;11:19864. doi: 10.1038/s41598-021-99357-y (PMC8494837; doi:10.1038/s41598-021-99357-y)
Supplement: Supplementary file 5 — Supplementary Information 5. [file 41598_2021_99357_MOESM5_ESM.pdf]

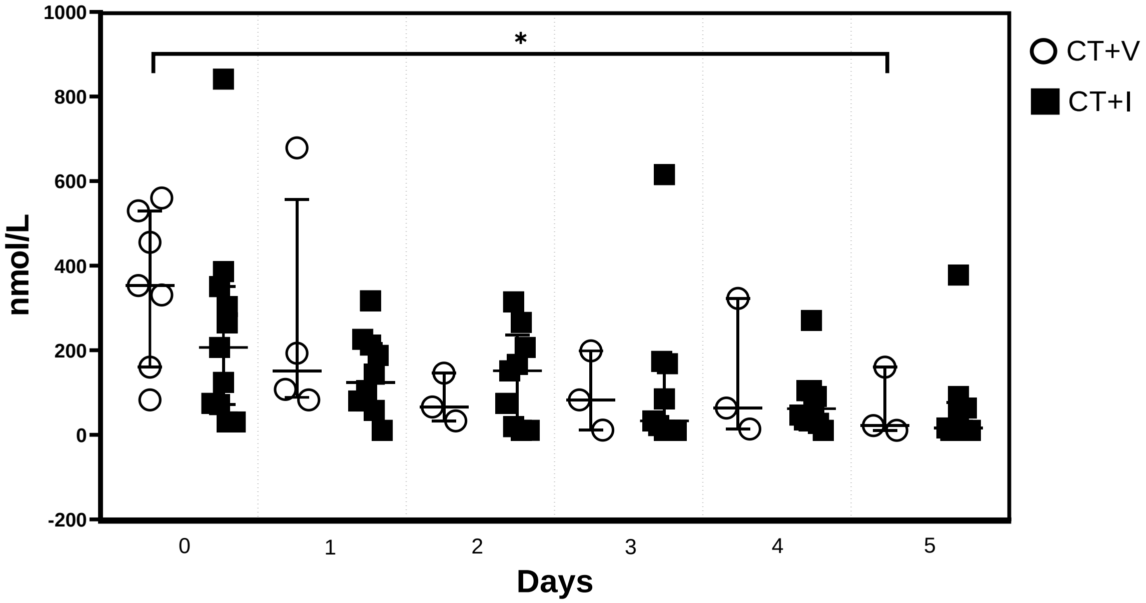

Figure S3. Effect of the immunomodulator over the concentration of plasmatic cortisol of puppies affected by CPE. Most of the puppies at their hospital admission showed high cortisol concentrations, which decreased over time in both groups. The Kruskal-Wallis test and Dunn’s posthoc test showed no statistical differences between cortisol levels of CT+V and CT+I groups, however, there are intragroup statistical differences in the CT+V group, between time 0 and 5, (*H*=32.82 , *df*= *104,1*; P≤ 0.0013). * P≤ 0.05. Each dot represents a patient. Median ± 5–95% confidence interval. CT= conventional treatment; I= immunomodulator; V= vehicle.
