# Supplementary material for: Increased survival in puppies affected by Canine Parvovirus type II using an immunomodulator as a therapeutic aid
Source: Sci Rep. 2021 Oct 6;11:19864. doi: 10.1038/s41598-021-99357-y (PMC8494837; doi:10.1038/s41598-021-99357-y)
Supplement: Supplementary file 6 — Supplementary Information 6. [file 41598_2021_99357_MOESM6_ESM.pdf]

Table S3. Inclusion, exclusion, and elimination criteria for the puppies.

Inclusion criteria	Exclusion criteria	Elimination criteria
• Any breed and sex under six months of age<br>• The appearance of clinical signs within a period no longer than 48 hours before consultation or diagnosis<br>• Minimum weight of 1 Kg<br>• Two or more clinical criteria for systemic inflammatory response syndrome<br>• Necessary hospitalization<br>• Owners willing to participate in the clinical trial with a	• Hospitalization under the observation of another veterinarian outside recruiting veterinary clinics/hospitals<br>• Necessary application of another immunomodulator or granulocytic colony-stimulating factor based on clinical evidence and at the discretion of a veterinarian	• Any adverse effects to administration of immunomodulator<br>• Suspended administration of immunomodulator<br>• Death within 24 hours after hospitalization

signed informed consent letter
